# 

**Published:** 1893-07-29

**Authors:** 


**OUR NEW OFFICES**

428, Strand, W.C., will henceforth be the Office of this Journal.

**Notices of Births, Deaths, and Marriages,** are inserted in this column at a charge of 2s. 6d. for an announcement not exceeding four lines.

### NOTICE TO CORRESPONDENTS.

All MS., Letters, Books for Review, and other matters intended for the Editor should be addressed THE EDITOR, THE LODGE, PORCHESTER SQUARE, LONDON, W.

The Editor cannot undertake to return rejected M.S., even when accompanied by stamped directed envelope.

**Notice to Advertisers and Subscribers.**

All Advertisements, Orders for Copies of THE HOSPITAL, and Business Communications should be addressed to The Publisher, *not to the Editor*, THE HOSPITAL, 428, Strand, W.C.

The Cost of Subscription, post free, is as follows:—Per week, 2½d.; per quarter, 2s. 6d.; per half-year, 5s.; per annum, 8s. 6d. Foreign:—Per annum, 10s. 8d.; payable, in all cases, in advance.

**"Burdett's Hospital Annual," 1893.**

Fourth year of publication. 700 pp. Boards, gilt lettering. A most complete guide to British, Colonial, Indian, and American Hospitals, Dispensaries, Nursing and Convalescent Institutions, and Asylums. Price 5s. Published by THE SCIENTIFIC PRESS (Limited), 428, Strand, W.C.

**NOTICE.—The Index for Vol. XIII. (ending March 1893) is on sale at the Office of this Journal, price 2½d., post free.**

### SCRAPS AND GLEANINGS.

Operations have been commenced in connection with the erection of the Dunfermline Cottage Hospital.

The State Board of Health of Boston has lately caused an examination to be made on the artificial ice which has been manufactured in America.

The chair of Pathology and Public Hygiene in the Medical School of Montreal has lately received from Sir Donald A. Smith the sum of 100,000 dollars.

Two amateur performances were held in the first week of July at the Exeter Theatre, with the object of raising the funds of the Hospital Saturday collection.

A conference of representatives of the sanitary authorities of London was held at the Court House, Marylebone Lane, respecting the hospital accommodation for scarlet fever and diptheria.

The Dewsbury Amateur Dramatic Society gave recently a donation of £8 towards the funds of the Infirmary, being the proceeds of two entertainments given in benefit of the institution.

The balance-sheet of the East Suffolk Hospital cannot be said to indicate an altogether satisfactory state of things, the year's expenditure exceeding the income in round figures by £700.

The attention of the Aldershot Local Government Board of Health has been called to the fact that the only accommodation provided for infectious diseases in the Farnham Union, is that of a ward in the workhouse.

A meeting in connection with the Pontypridd Cottage Hospital was recently called relating to the representation of working classes on the committee, It was suggested that ten should serve on a committee of fifteen.

Another outbreak of small-pox having occurred in the Hereford district, the Board of Guardians met to push forward the final approval of the architect's plans for the proposed infectious hospital in that locality.

On June 30th a reception of subscribers and friends was held at the Princess Alice Memorial Hospital, Eastbourne, when the various wards, &c., were inspected. Light refreshments being served in the dining and other rooms.

The total amount received by the Hospital Saturday collection at Newcastle was £140; in Tunstall the amount thus collected was £168. At Newcastle the distress in the town accounts for the smallness of the sums received.

The Archbishop of Canterbury, who entertained recently in the grounds of Lambeth Palace a number of blind persons at tea, upon leaving, after shaking hands with his guests, presented a bunch of flowers to each.

The twenty-sixth annual meeting in connection with the Belgrave Hospital for Sick Children took place last month. The Committee appealed for further aid, failing which they would be compelled to close one of the wards.

Resolutions were passed at a meeting of the subscribers to the National Dental Hospital, thanking the Dowager Lady Howard de Walden for her munificent gift of the new buildings, which are to be opened in October next.

A conference was held at Middleton St. George, on the 2nd July, for the purpose of considering the desirability of establishing a permanent convalescent home in the country for the benefit of the workmen and poor at Teeside.

At the annual festival dinner in aid of the funds of the Earlswood Asylum, the Chairman stated that out of the total number of inmates 199 of the males and 41 of the females were at the present moment employed in remunerative pursuits.

In presenting the fifth annual report of the Victoria Jubilee Hospital at Swaffham (Norwich), the sub-committee of management have again to congratulate the subscribers and donors on the financial position of the same, the balance in hand amounting to £98.

At a meeting held this month by the Local Government Board, on the administration of the Islington Workhouse, Mr. Musto, the master, was severely centured for the severity of his conduct in two cases where the inmates had deserved less punishment at his hands.

At the annual meeting of the subscribers of the Retford General Dispensary and Cottage Hospital, the treasurer presented accounts which showed that the receipts for the year, including a balance from the previous year amounted to £517, leaving £235 to the good.

A public meeting was held on June 29th, convened by the Upper Norwood Ratepayers' Association in the hall of the Royal Normal College for the Blind, for the purpose of protesting against the establishment of a Fever Hospital at Grange Wood, near Beulah Hill.

Six hundred Hospital Saturday Fund collection boxes from the central districts were opened at the Drill Hall, Farringdon-road, under the direction of Mr. Nicolls, City manager of the Holborn branch of the City Bank, and were found to contain £1,431 in gold, £715 in silver, and £597 in coppers.

The annual demonstration for the benefit of the funds of the Huddersfield Infirmary was held at Deighton recently; a procession of trades took place, accompanied by several brass bands. After the payment of expenses it was expected a substantial sum would remain for the Infirmary funds.

The net proceeds of the donations received for the Royal Hospital, Richmond, amount to £1,749, of which sum total the Duke and Duchess of Teck and Princess May raised by their own exertions £812, in addition to a promised donation of £[illegible],000 by Mr. J. D. Charrington to be devoted to founding the Hilda May Cot.

Eighteen friendly and trade societies took part in a demonstration on the 2nd July at Hartlepool with the object of augmenting the funds of the local hospital. It was stated at the meeting that the death-rate in the institution was only 4 per cent.; 122 operations had been performed, and there had only been three deaths. The general efficiency of the institution was further commented upon.

### MEDICAL VACANCIES AND APPOINTMENTS.

**APPOINTMENTS.**

G. M. Arkle, L.S.A.Lond., appointed Assistant Surgeon to the South Dispensary, Liverpool. R. H. F. Bostock, L.R.C.P., L.R.C.S.Edin., appointed Medical Officer of Health to the Norton Urban Sanitary District of the Malton Union. A. E. Bridger, B.A., M.D., F.R.C.P.Edin., appointed Physician to the St. Pancras and Northern Dispensary. G. Brown, M.D., L.F.P.S.Glasg., appointed Medical Officer of Health for the Colchester Urban Sanitary District of the Colchester Union. T. L. Bryan, M.B.Dunelm, appointed Medical Officer to the Brough District of the Kirkby Stephen Union. A. Cameron, M.D., C.M.Glas., reappointed Medical Officer of Health for the Caistor Rural Sanitary District. T. M. Cann, M.R.C.S.Eng., L.M., appointed Medical Officer for the First Division of the Third District of the Newhaven Union. H. F. T. Chambers, L.R.C.P., L.R.C.S.Edin., appointed Medical Officer for the Wareham District of the Wareham Union. W. F. Cleaver, L.R.C.P.Lond., M.R.C.S.Eng., appointed Medical Officer of Health to the Phillac Urban Sanitary District. H. C. De Renzi, M.R.C.S.Eng., L.R.C.P.Lond., late Senior House Surgeon, appointed Resident Obstetric Assistant to the Westminster Hospital. T. W. Eden, M.D.Edin, C.M.Edin., appointed Pathologist to the Chelsea Hospital for Women. A. E. Giles, M.D.Lond., M.R.C.P.Lond., B.Sc., appointed Physician to Out-Patient to the Chelsea Hospital for Women. L. Hill. M.B., appointed Assistant Professor of Physiology at University College, London. A. Hind, L.R.C.S.Edin., L.M., L.S.A., reappointed Medical Officer of Health for the South Molton Urban Sanitary District. F. D. Holmes, B.A.R.U.I., L.R.C.P., L.R.C.S.Edin., appointed Medical Officer for the Breadsall District of the Shardlow Union. B. Keane, L.R.C.P., L.R.C.S.Edin., appointed Medical Officer for the Western District of the Mile End Union. A. King, L.D.S., appointed Honorary Dental Surgeon to the Royal Surrey County Hospital, Guildford. S. H. Lee, M.B., B.C., B.A.Cantab., M.R.C.S., L.R.C.P., late House Surgeon, late House Physician, appointed Obstetric House Physician at Middlesex Hospital. H. H. Littlejohn, M.A., M.B.Edin., C.M., F.R.C.S., reappointed Medical Officer of Health for Sheffield. A. Macbeath, L.R.C.P., L.R.C.S.Edin., appointed Medical Officer for the District of Unst. A. S. McCausland, M.D.Brux., L.R.C.P.Lond., M.R.C.S., appointed Certifying Factory Surgeon for the Swanage District. A. G. Mackenzie, M.R.C.P., F.R.C.S.Edin., reappointed Medical Officer for the Third Sanitary District of the Church Stretton Union. H. H. Mills, M.R.C.S., L.R.C.P., appointed an Assistant Demonstrator in Anatomy at the Westminster Hospital Medical School. A. G. Minshall, L.R.C.P.Lond., M.R.C.S.Eng., appointed Senior Assistant Medical Officer to the St. Pancras Parish Infirmary. I. Mossop, F.R.C.S., L.R.C.P.Edin, appointed Honorary Medical Officer to the Bradford Children's Hospital. C. Munden, M.R.C.S., L.S.A., reappointed Medical Officer for 3 c Sanitary District of the Langport Union. T. H. Openshaw, M.S., M.B.Durh., F.R.C.S.Eng., appointed Lecturer on Anatomy at the London Hospital Medical School. R. E. F. Pearse, L.R.C.P.Lond., M.R.C.S.Eng., reappointed House Surgeon Jagersfontein Hospital, Orange Free State, South Africa. W. R. Pollock, M.B., B.C.Camb., M.R.C.P.Lond., appointed Assistant Obstetric Physician to the Westminster Hospital. H. W. Pooler, L.R.C.P., M.R.C.S., L.S.A., appointed Honorary Administrator of Anæsthetics to the Dental Hospital, Birmingham. A. W. Port, M.B., B.S., M.R.C.S., appointed House Surgeon to the Monkwearmouth and Southwick Hospital Sunderland. C. Pound, L.R.C.P.Lond, L.S.A., appointed MedicalOfficer for the Odiham District of the Hartley Wintney Union. E. S. Robinson, L.R.C.P.Lond., M.R.C.S.Eng., appointed Medical Officer of Health for the Stourport Urban Sanitary District of the Kidderminster Union. W. Rushton, L.D.S.R.C.S.Eng., appointed Dental Surgeon to the Hospital for Consumption and Diseases of the Chest, Brompton. P. de Santi, F.R.C.S., appointed Demonstrator of Anatomy at the Westminster Hospital Medical School. A. C. F. Smith, L.R.C.P. and S.Edin., appointed Assistant House Surgeon to the Royal Albert Hospital and Eye Infirmary, Devonport. H. F. Stokes, M.R.C.S.Eng., L.R.C.P.Lond., appointed Medical Lecturer at the Church Missionary College, Islington. B. T. Stokoe, M.B., B.S.Durh., appointed Medical Officer of Health for the Borough of Gateshead. R. P. Williams, L.R.C.P., L.M., L.R.C.S.Edin., appointed Medical Officer to the Holyhead Post Office employes.

### MEDICAL APPOINTMENTS.

**ROYAL FREE HOSPITAL, Gray's Inn Road.**

### MEDICAL VACANCIES AND APPOINTMENTS—(*continued*)

W. A. Wills, M.D.Lond., appointed Lecturer on Elementary Medicine at the Westminster Hospital Medical School. P. M. Yearsley, L.R.C.P.Lond., M.R.C.S.Eng., appointed an Assistant Demonstrator in Anatomy at the Westminster Hospital Medical School. W. Loynd, M.R.C.S. Eng., L.R.C.P.I., reappointed Medical Officer of Health to the Church Local Board. L. W. Darra Mair, M.D.Lond., D.P.H.Lond., M.R.C.S., L.R.C.P., appointed Medical Officer of Health of the Croydon Rural Sanitary Authority. G. T. K. Maurice, L.R.C.P.Lond., M.R.C.S.Eng., appointed Medical Officer for the First District and the Workhouse of the Marlborough Union. J. Mudge, L.R.C.P., L.M.Edin., M.R.C.S., reappointed Medical Officer for the Phillack Urban Sanitary District of the Redruth Union. C. Ogden, L.R.C.P., L.M.Edin., M.R.C.S.Eng., reappointed Medical Officer of Health for the Milnrow Urban Sanitary District of the Rochdale Union. A. M. Palmer, L.R.C.P., L.M.Edin., M.R.C.S.Eng., appointed Medical Officer of Health to the Whittington Local Board. H. M. Richards, M.B., B.S.Lond., M.R.C.S.Eng., appointed Medical Superintendent to the City Hospital, Birmingham. A. Roberts, F.R.C.S. Edin., M.R.C.S.Eng., reappointed Assistant Surgeon to the Royal Berkshire Hospital. Mr. C. A. Ross, appointed Medical Officer for the Tenbury District and the Workhouse of the Tenbury Union. Mr. Thompson, appointed Medical Officer for the Abbotsham District of the Bideford Union. R. G. Tucker, L.R.C.P.Edin., M.R.C.S.Eng., appointed Medical Officer for the Third District of the Dartford Union. H. F. Waterhouse, M.D., C.M.Edin., F.R.C.S.Eng., appointed Surgeon to Out-patients, Victoria Hospital for Sick Children. G. W. Weir, M.D., M.Ch., D.P.H.Camb., reappointed Medical Officer of Health for Jarrow-on-Tyne. A. H. Whicher, M.R.C.S.Eng., L.S.A., appointed Medical Officer of Health for the Midsomer Norton Urban Sanitary District. Dr. Wiggins, appointed Deputy Medical Officer and Public Vaccinator to the Bacup District of the Haslingden Union.

### VACANCIES.

#### Resident Medical Officers.

Chesterfield and North Derbyshire Hospital and Dispensary, Chesterfield. House Surgeon. Salary, £100 per annum. Assistant House Surgeon. Salary £50, with board, apartments, and laundress for each. Applications to the Secretary, by August 4th.

General Hospital for Sick Children, Pendlebury, Manchester. Junior Resident Medical Officer, doubly qualified. Salary £80 per annum, with board and lodging. Applications to the Chairman of the Weekly Board by July 29th.

Great Yarmouth Hospital. House Surgeon, doubly qualified. Salary, £90 per annum, with board and lodging. Applications to R. F. E. Ferrier, Hon. Secretary, by August 1st.

Lewes Dispensary and Infirmary and Victoria Hospital, High Street, Lewes. Resident Medical Officer, doubly qualified. Salary, £100 per annum, furnished apartments, coal, gas, and attendance. Applications to the Honorary Secretary, Mr. Reginald Blaker, Lewes, Sussex, by July 31st.

Liverpool Children's Hospital. *Locum tenens* House Surgeon for one month. Board, rooms, and washing. Applications to the House Surgeon.

Manchester Royal Infirmary. Resident Medical Officer, not less than 25 years of age and unmarried; doubly qualified. Appointment for one year, but holder of office may be re-elected. Salary, £150 per annum, with board and residence. Applications to the Chairman of the Board by July 29th.

New Hospital for Women, 144, Euston Road, N.W. Female Clinical Assistant for In-patient Department; fully qualified. Applications to M. M. Bagster, Secretary, by July 28th.

Royal South Hants Infirmary, Southampton. Assistant House Surgeon. No salary, board and rooms provided. Applications to T. A. Fisher Hall, Secretary, by July 31st.

Whitehaven and West Cumberland Infirmary, Whitehaven. House Surgeon; doubly qualified. Salary £120 per annum, and £30 per year for dispensing, with furnished apartments and attendance. Applications to Tyson Kitchen, Secretary, by July 29th.

#### Non-Resident Medical Officers.

Grosvenor Hospital for Women and Children, Westminster. Assistant Physician and Anæsthetist. Applications to the Secretary by July 29th.

Islington Dispensary, 303, Upper Street, N. Dispenser; not over 35 years of age. Salary 40s. per week. Applications to the Honorary Secretary.

Portumna Union, Portumna Dispensary No. 2. Medical Officer. Salary £40 per annum, with £5 as Medical Officer of Health, registration and vaccination fees. Applications to Mr. Laurence Taylor, Honorary Secretary. Election on August 5th.

Royal Free Hospital, Gray's Inn Road, W.C. Assistant Physician. Applications to the Secretary by July 31st.

Tralee Union, Tralee Dispensary. Apothecary. Salary £20 per annum. Applications to Mr. W. Denny, Honorary Secretary. Election on August 6th.

